# Bilateral Peripapillary Retinal Nerve Fiber Layer Myelination in a 26-Year-Old Man

**DOI:** 10.7759/cureus.9960

**Published:** 2020-08-23

**Authors:** Ejike Egbu

**Affiliations:** 1 Eye Center, Lily Hospitals Limited, Warri, NGA

**Keywords:** retinal nerve fiber layer, myelination, oligodendrocytes, lamina cribrosa, optic nerve, peripapillary

## Abstract

Myelination is an uncommon condition that rarely causes visual impairment. We report a case of a young man with bilateral peripapillary nerve fiber layer myelination and visual impairment. The patient's complaints, clinical history, and systemic review were noted. Visual acuity test, objective and subjective refraction, ocular motility, fundus examination, optical coherence tomography, and low vision test, were performed. We compared our findings with published articles. We suggest that optic neuropathy was the cause of visual impairment in the patient.

## Introduction

Myelin was first mentioned in the 19th century by the German pathologist Rudolf Virchow and further described in the 1870s by the French physician Louis-Antoine Ranvier. It is made up of lipids and proteins and wrapped around the axons of neurons. In the central nervous system, myelin sheaths are formed by oligodendrocytes, while in the peripheral nervous system, it is produced by the Schwann cells [1]. Myelin shows a lipid and a protein-dependent birefringence when viewed under polarized light. It shows three peaks that correspond to a bimolecular lipid leaflet and an adjacent protein layer during low-angle X-diffraction studies [2]. It is characterized by a high lipid proportion of 70-85% and about 15-30% of protein. In the central nervous system (CNS), its primary protein composition is the proteolipid protein, which takes about 50% of mass while in the peripheral nervous system (PNS), it is composed of majorly protein zero with about 80%of mass [3, 4]. Myelin serves as an electrical insulator by facilitating rapid impulse conduction in axons, thereby making them "hop" unlike the unmyelinated ones with continuous impulses [3, 5].

Myelination of the retinal nerve fiber layer (RNFL) is a congenital, non-progressive lesion appearing as white patches on the retina. They are generally sporadic and found in 0.57-1.0% of the population and associated with myopia, amblyopia, and strabismus. The mechanism of myelination of RNFL is unclear - it begins in the eight-month of intrauterine life and is thought to occur when the migration of oligodendrocytes occasionally extends anterior to the lamina cribrosa along the nerve fibers of the optic nerve head to the sensory retina [6, 7]. Hence, any factor that affects the anatomical structure and function of the lamina cribrosa may lead to the myelination of RNFL. Myelinated RNFLs are mostly unilateral; 7.7% of cases are bilateral, and patients with them are mostly asymptomatic. In affected eyes, RNFL myelination may be continuous (33%) or discontinuous (66%) with the optic nerve [7]. Ramkumar et al. also noted that retinal myelination could have multiple distinct lesions, and these have been found in about 12% of patients. Myelination of the RNFL has been associated with the following conditions: high myopia and reduced retinal thickness, pale neuroretinal rim, neurofibromatosis, Down's syndrome, and craniofacial dystosis. It can also be mistaken for other conditions such as cotton-wool spots, retinal infiltrate, retinal artery occlusion, and even leukocoria [7-9].

## Case presentation

The patient is a 26-year-old man who presented with complaints of poor vision in both eyes since childhood, which has used several spectacles but to no avail. He is a receptionist in a retail shop and not exposed to any toxic substances such as lead. There was no history of the monotonous diet of cassava products, consumption of alcohol, or smoking. There were no associated symptoms nor significant findings in the review of systems.

Unaided distance visual acuity was 1/60 bilaterally, and there was no improvement with refraction. Ocular motility was full in all directions of gaze, and corneal reflex was central on the pen torch examination. The anterior segment was normal bilaterally. The pupillary reaction was sluggish, and there was no relative pupillary defect (RAPD). Intraocular pressure (Goldman) was 18mmHg and 22mmHg in the right and left eyes, respectively. The optical coherence tomography (OCT) findings showed in the right - pale disc, cup to disc ratio of 0.3x0.2, normal macula, normal vessels, and myelination of the retinal nerve fiber layer. The left showed pale disc, cup to disc ratio of 0.5x0.3, normal macula, normal vessels, and myelination of the retinal nerve fiber layer.

Objective refraction was Plano/-0.50x139 and Plano/-0.50x167 for right and left eyes, respectively. Keratometry was K1: 42.75D, K2: 43.50D for the right eye and K1: 43.00D, K2: 44.00D for the left eye. The axial length was 23.84mm and 23.94mm right and left eye, respectively. Low vision aids improved his distance visual acuity to 6/12 in both eyes with a handheld telescope (X8) and with a stand magnifier (X12), his near vision improved to N.10.

**Figure 1:**
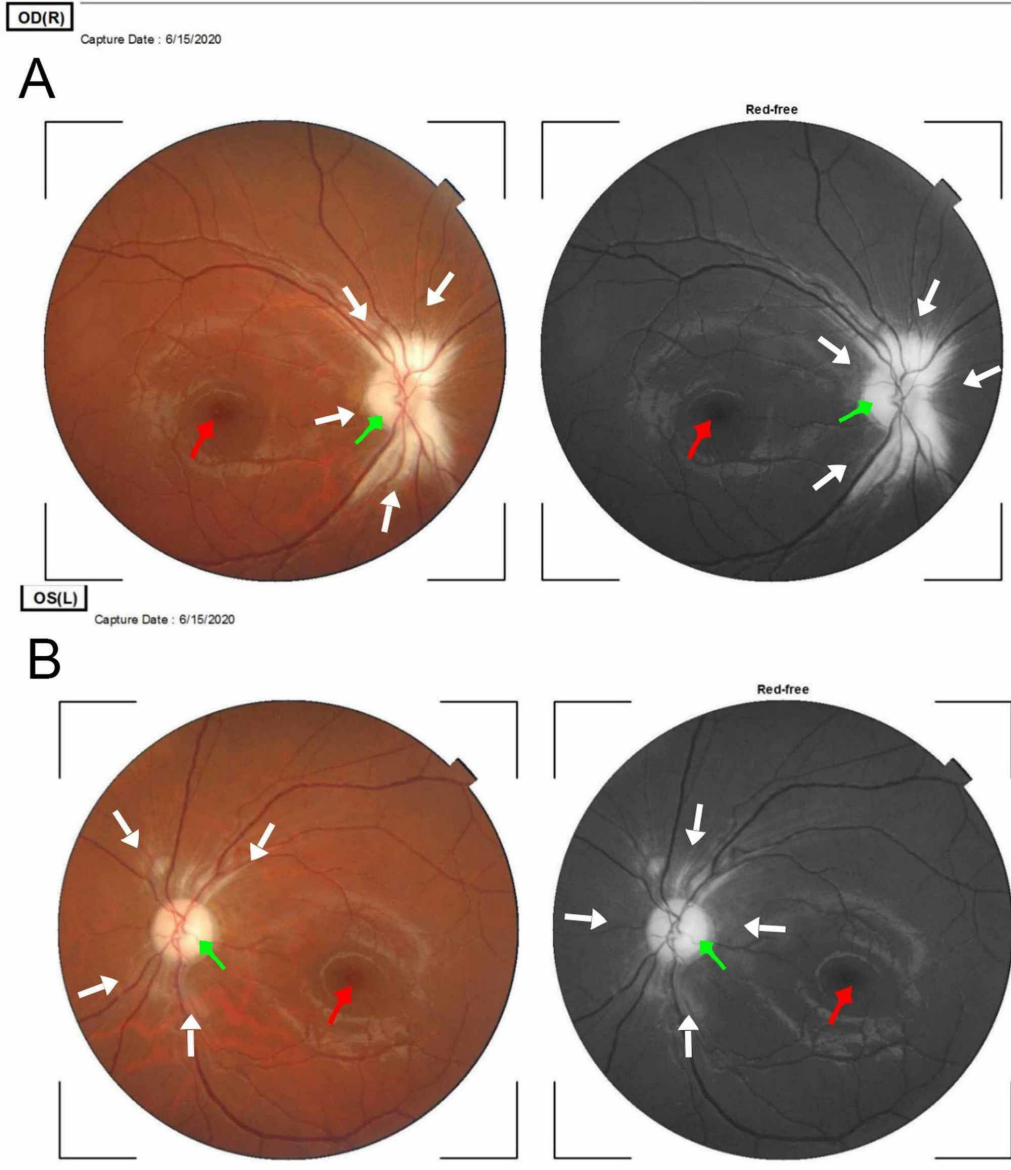
Fundus photographs (colored and red-free) of the right (A) and left (B) eye Panes A and B are fundus photographs of the right and left eye, respectively, showing myelination of the RNFL. The lesion is contiguous with the optic disc in both eyes. The white arrows point to the myelinated retinal nerve fiber layer in both eyes. There is also palor of the neuroretinal rim bilaterally, indicated by the green arrows. The lesion in the right eye is larger than the left. The red arrows point to the normal appearance of the fovea centralis in both eyes. RNFL - retinal nerve fiber layer

**Figure 2:**
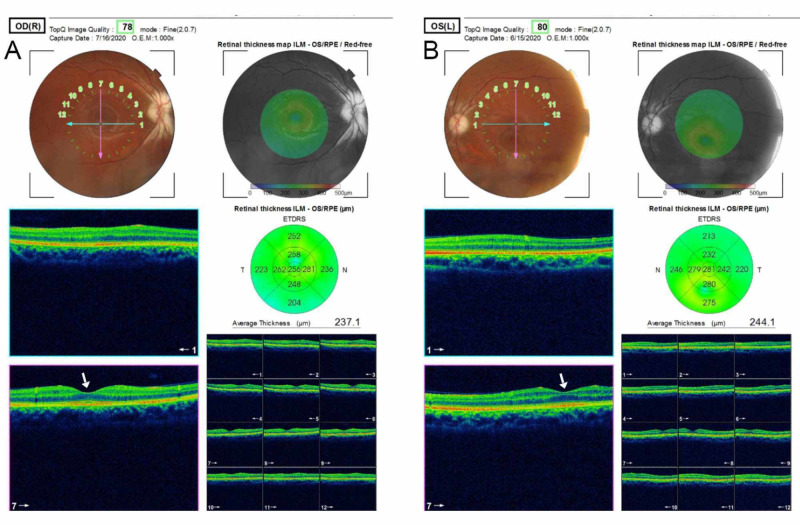
The optical coherence tomography of the macula of the right (A) and left eye (B) The fovea is indicated by the white arrows and appears normal in both eyes.

The findings on optical coherence tomography are in Figures 1 and 2.

## Discussion

Myelination of the retinal fiber layer is a rare condition that may be found in patients during funduscopic examination for other eye conditions. It is more commonly an asymptomatic, congenital condition as such, only a few cases are diagnosed. This condition has not been shown to have any sex or racial predilection. It may be associated with some syndromes; however, the index case did not have any associated syndrome, and there was no notable finding in the review of systems. This report is peculiar because the myelination of the retinal nerve fiber layer was associated with atrophy of the optic disc bilaterally and low vision.

Myelination of nerves profers the advantage of increased conduction of action potential along axons of nerves through a series of mechanisms that have not been fully understood [10]. Some of these mechanisms include activation of extrasynaptic receptors and sensing neuronal activity through potassium channels [11]. The interaction between oligodendrocytes and neurons ultimately determines the structure and function of neuronal circuits [12] The index patient developed low vision, which is suggestive of a congenital lesion, which affected optimal visual development in infancy and childhood. The mechanism of low vision is unclear and may be due to a defective transmission of an electrical impulse along the retinal nerve fiber layers caused by myelination. A finding of bilateral visual acuity of 1/60, clear visual axis, and full ocular motility in all directions of gaze are highly suggestive of optic neuropathy in the absence of a macular lesion. Low vision in this patient may also be due to some level of optic nerve hypoplasia as the findings on biometric evaluation and refraction were essentially within normal limits. The funduscopic findings of this patient showed that the myelination was along the superior and inferior arcades of the retina and contiguous with the optic disc bilaterally, the neuroretinal rim was pale, and there was no apparent macula lesion bilaterally (see Figures 1-2). It is also possible that myelinated fibers blur retinal images and induce visual deprivation leading to low vision. The most probable cause of low vision is optic atrophy due to a disruption of the vascular supply to the optic nerve. This supports the findings of Holló et al., who found that myelination affects peripapillary angioflow density and caused decreased vessel density in the optic nerve head in a study of the Influence of myelinated retinal nerve fibers on retinal vessel density measurement with AngioVue® OCT angiography [13].

This case is similar to that reported by Gottfried Jean-Louis et al. in a seven-year-old male diagnosed with myelination of the retinal nerve fiber layer with associated optic nerve drusen in the right eye, and the subsequent appearance of a similar lesion in the left eye at 14 years. The lesions were described as acquired and progressive but did not lead to loss of vision as visual acuity remained 6/6 bilaterally [9]. This was not the case in the patient described in this report who developed a low vision with a visual acuity of 6/60 bilaterally. Also, unlike the patient in this report, there was a preservation of the neuroretinal rim of the optic discs with a pink appearance in both eyes, hence the good visual acuity. This further suggests that optic disc drusen may not affect the vascular supply to the optic nerve leading to optic atrophy.

In this case, optic atrophy was a result of the damage of the axons of retinal ganglion cells, most likely due to myelination [14]. The mainstay of management of low vision is the use of low vision devices. Low vision in a young adult causes challenges with activities of daily living such as reading, cooking, communicating, and sports [15]. It may also lead to psychological problems of low self-esteem and depression because these devices are not cosmetically appealing, hence the need to provide counseling, psychological, and social support to the patient. Finally, there is a need for periodic follow up to evaluate the size of the lesion and change in visual function.

## Conclusions

Myelination of the retinal nerve fiber layer is an uncommon condition of the retina that can result in optic atrophy and impairment of vision. The mechanism of low vision, in this case, was probably due to a disruption of neurotransmission along the myelinated nerve fiber layer that forms the optic nerve or a compromise of the vascular supply to the optic disc leading to atrophy of the optic nerve.
